# Radiotherapy for Lung Cancer: An Unrecognized Cause of Lung Torsion

**DOI:** 10.7759/cureus.79936

**Published:** 2025-03-02

**Authors:** Savindika Nawarathna, Roshni Mava, Dipanjan Banerjee, Samir Guglani, Garrett McGann

**Affiliations:** 1 Radiology, Severn School of Radiology, Bristol, GBR; 2 Clinical Oncology, Cheltenham General Hospital, Cheltenham, GBR; 3 Radiology, University Hospitals Bristol and Weston, Bristol, GBR; 4 Radiology, Cheltenham General Hospital, Cheltenham, GBR

**Keywords:** lung cancer, lung torsion, oncology, radiology, radiotherapy

## Abstract

Lung torsion is an exceptional occurrence, usually requiring urgent surgery because of vascular compromise. Because of the rarity and non-specific symptoms, it often requires clinicians to have a high degree of suspicion to make an early diagnosis of lung torsion. Here we present a unique case of lung torsion. The patient was a 73-year-old lady who was diagnosed with adenocarcinoma of the lung. She was treated with radiotherapy, which resulted in complete remission. However, her follow-up imaging showed torsion of the right lung. Given her lack of clinical symptoms, she was managed conservatively. This is a unique case and possibly the first reported case of lung torsion following radiotherapy. The exact mechanism through which radiotherapy causes lung torsion remains unclear and may be addressed by future research.

## Introduction

Numerically, the most common causes of torsion are trauma and post-surgery. Fundamentally, anything that can distort the normal architecture of the lungs may contribute to the development of lung torsion [1]. A few cases of spontaneous occurrences with an underlying pulmonary abnormality have also been reported in the literature [2]. The mechanism of development of lung torsion varies based on the etiology. Qaqish et al., for example, demonstrated a case of spontaneous lung torsion due to a densely consolidated right upper lobe, nearly complete oblique fissure, and associated pleural effusion, causing parenchymal rotation around the bronchovascular pedicle [3]. Early diagnosis of lung torsion remains challenging due to variable presentation, hence a high degree of suspicion is often necessary for a prompt and early diagnosis to avoid complications such as hemorrhagic infarction, gangrene, or death [4-5]. Here, we present a unique case of a patient diagnosed with lower lobe torsion post-radiotherapy detected on a follow-up computed tomography (CT) scan at the lung multidisciplinary meeting.

## Case presentation

**Figure 1:**
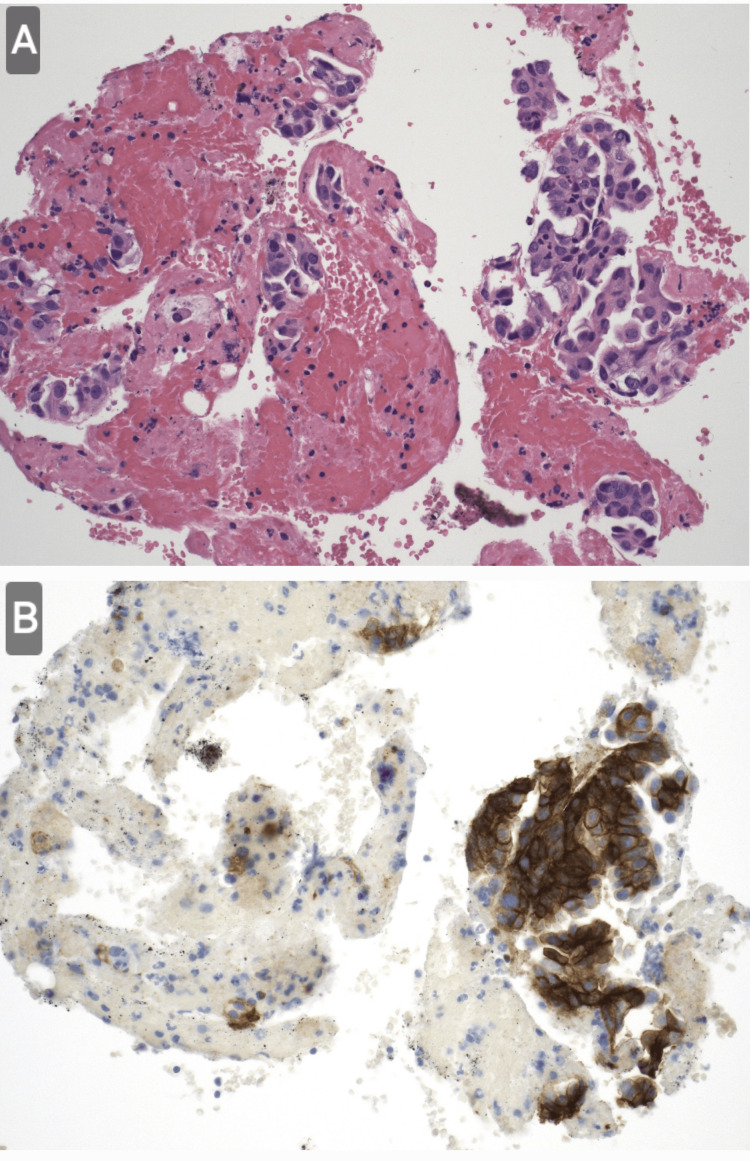
Scattered clusters of cytologically malignant epithelial cells are present arranged in micropapillary nests. (A) Hematoxylin and eosin staining shows multiple clusters of tumor cells in a limited area, against the background of inflammatory cells. (B) It appeared positive on the PD-L1 staining. Other immunohistochemistry (ALK/ROS/NTRK) was negative.

A 73-year-old lady presented with a long history of bilateral chest wall discomfort, exertional breathlessness, and chronic fatigue. She was found to have a left lung opacification on chest radiograph, which prompted a computed tomography scan and positron emission tomography-computed tomography (PET-CT) scan, which confirmed an avid 53 mm left upper lobe mass with associated hilar and mediastinal lymphadenopathy staging the disease as T3N2M0. An endobronchial ultrasound scan and biopsy (EBUS) sampling of station 11L confirmed lung adenocarcinoma metastasis (Figure 1), and molecular testing confirmed 60% PD-L1 positivity.

**Figure 2:**
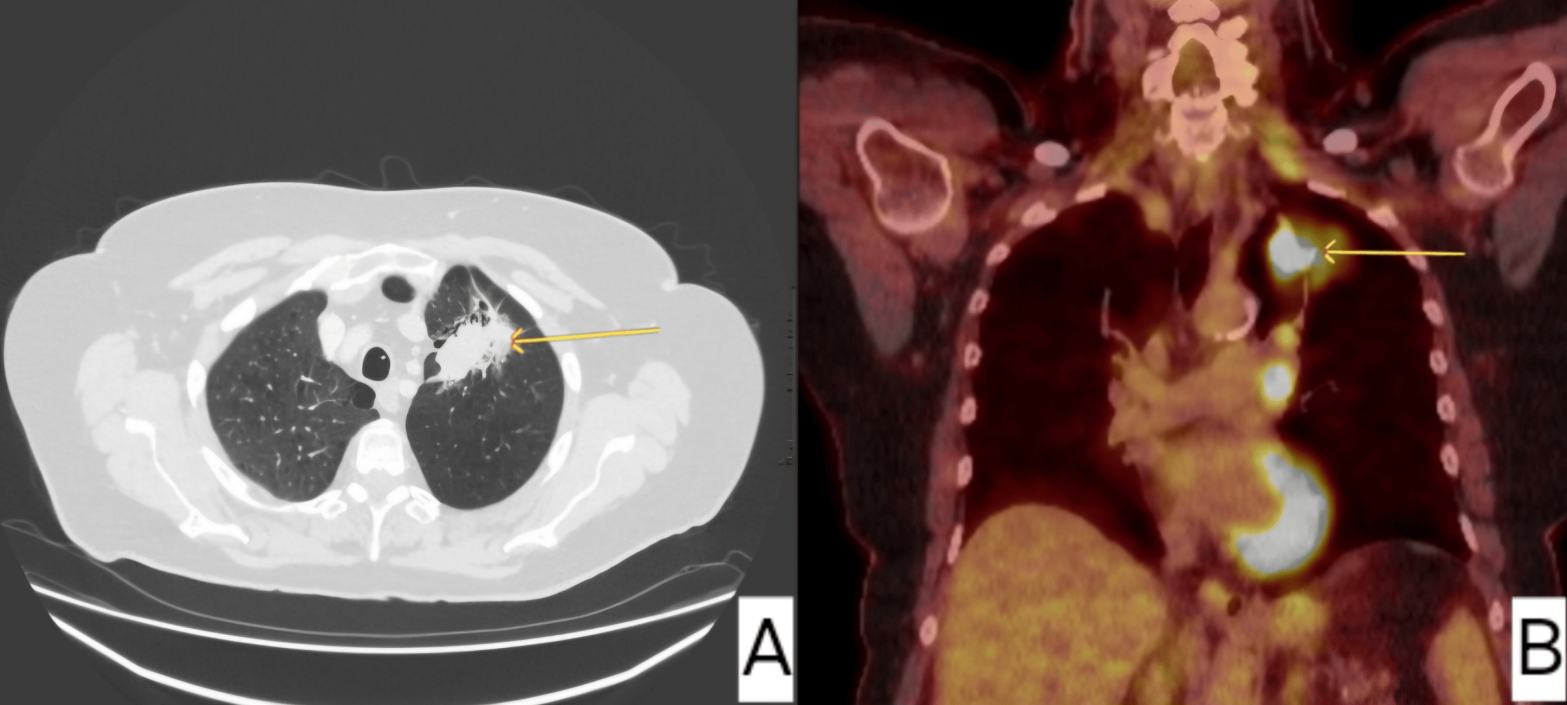
Axial images of speculated left apical mass, also demonstrating significant avidity in PET. (A) Axial images demonstrated a left lung spiculated mass. (B) Subsequent PET imaging showed an avid 53 mm left upper lobe mass with associated hilar and mediastinal lymphadenopathy staging the disease as T3N2M0.

**Figure 3:**
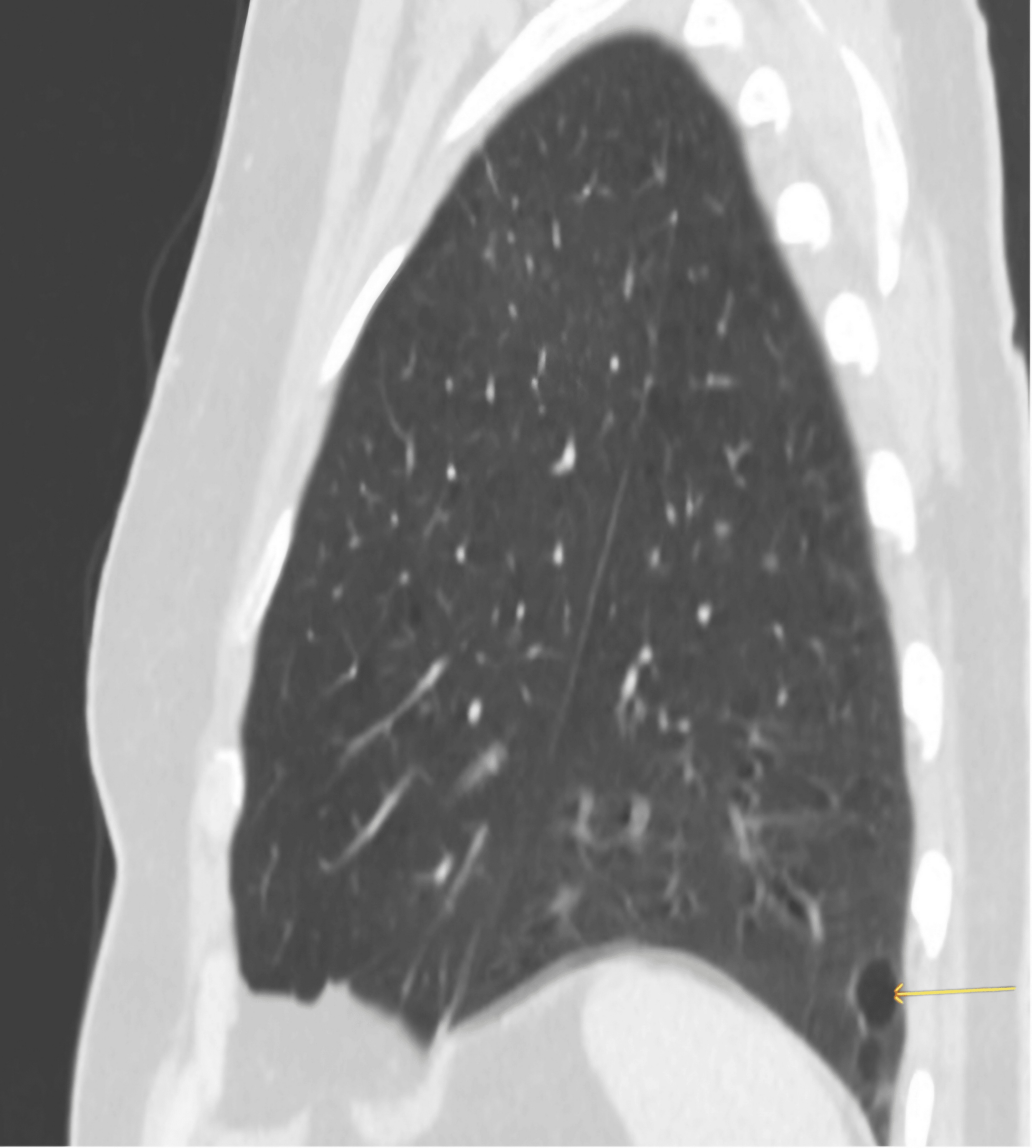
Post radiotherapy imaging showed complete response to treatment. It also showed two small bullae seen in the post aspect of the lower lobe.

Her lung function at this point was 51% forced expiratory volume (FEV1) (1.21) and 73% diffusing capacity of the lungs for carbon monoxide (DLCO). She consented to radical treatment with four weeks of radiotherapy dosed at 55 Gy/20 with concurrent chemotherapy. Her initial scan (Figure 2) post radiotherapy showed a limited response to treatment, but her subsequent CT scan (Figure 3) in a year showed that there was no tumor visible on axial images but there was apparent collapse of the left lung base.

**Figure 4:**
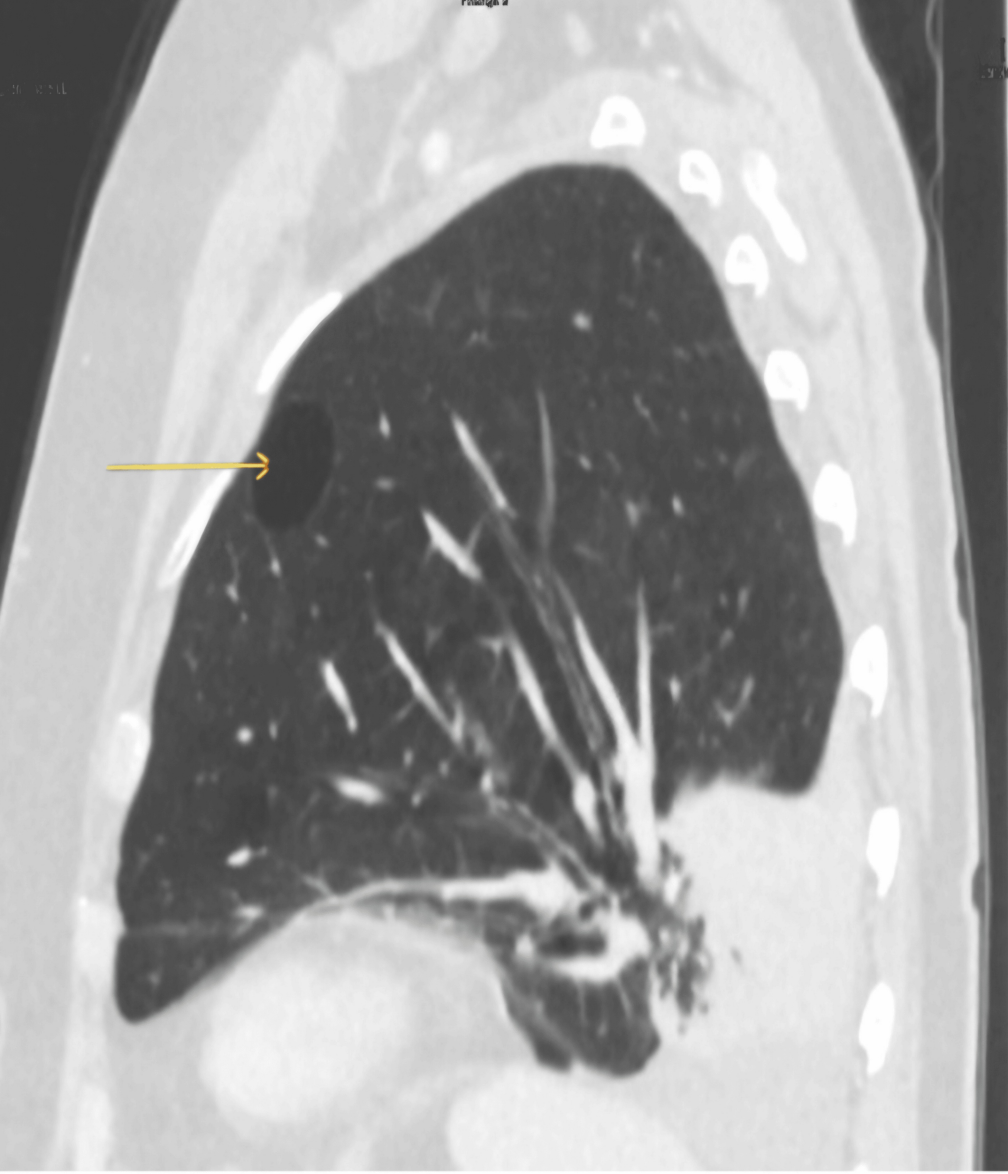
Follow-up imaging after 12 months since previous imaging demonstrated the migration of the emphysematous bullae anteriorly and superiorly towards the apex of the lung. There was also an associated left lung base collapse.

**Figure 5:**
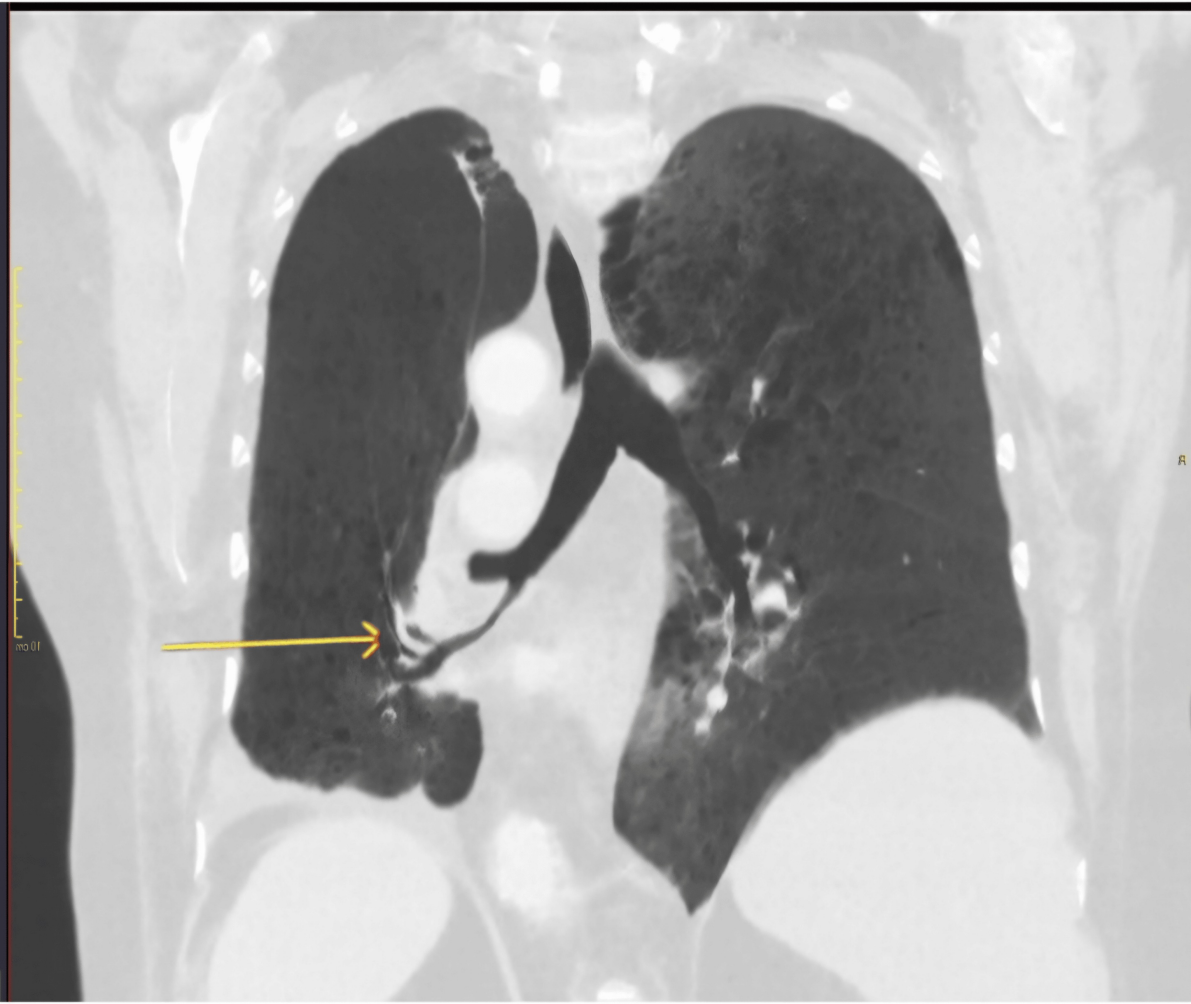
Coronal views on lung window shows tapering and curved orientation of the lower lobe segmental bronchi and pulmonary vessels of the right lung indicating twisting of the lobe. Lung torsion with 180-degree rotation was diagnosed.

Five months later, she had a follow-up scan (Figures 4, 5). Coronal views showed upward curving of the lower segmental bronchi and pulmonary vessels. The two small bullae initially seen in the post aspect of the lower lobe seemed to have relocated to the upper zone anteriorly. Lung torsion with 180-degree rotation was diagnosed. The patient was clinically well; hence, it did not warrant surgical referral.

## Discussion

A literature review from 1950 to 2014 showed that lung torsions are a rare occurrence, with an incidence of 0.089% to 0.4% [6]. It is a life-threatening emergency that warrants prompt diagnosis and management. The review showed that lung torsions occurred commonly in patients post-operatively, after trauma, and in some instances spontaneously. It also found that 74.4% occurred after a right upper lobe lobectomy, and the common site is the middle lobe [6]. The pathophysiology remains unclear to date, but the predisposing factors include a complete interlobar fissure, an absence of adhesions, a narrow middle lobe hilum, and extensive mobilization at surgical dissection of intrathoracic attachments and the inferior pulmonary ligament [7]. Various authors have attempted to explain the pathophysiology of lung torsion and the subsequent development of infarction, including the suggestion of venous outflow obstruction potentially leading to the development of ischemia [8,9]. While non-specific clinical symptoms such as dyspnoea, fever, and chest pain might be seen at presentation, imaging, namely a computed tomography scan plays a vital role in diagnosis [10,11]. The main radiographic findings include an unusually located collapse or consolidated lobe, hilar displacement not in keeping with a lobe that appears to be atelectatic, change of position of the pulmonary vasculature with rapid opacification of an ipsilateral lobe, sequential change of position of the opacified lobe, bronchial distortion, lobar air trapping and primary or secondary signs of lobar collapse [2]. Bronchoscopy can also be used to diagnose classic fish mouth orifice or bronchial stenosis seen during the procedure, but it is mostly followed by a scan [12]. Early detection is essential to avoid complications such as hemorrhagic infarction, gangrene, or death [5]. Emergency lobectomy is recommended for all cases of lung torsion, as salvage by detorsion runs the risk of ischemia-reperfusion injury [13]. This wasn’t the case with our patient, who was asymptomatic, and so did not require surgical management. In comparison, conservative management runs the risk of various outcomes, like in the case reported by Lashari et al. we believe that conservative management is the most appropriate decision [14].

## Conclusions

We present a case of lung torsion caused by radiotherapy diagnosed by sequential computed tomography scan and managed conservatively due to an asymptomatic patient. To our knowledge, this is the first report of pulmonary torsion caused by radiotherapy. Further research might be required to explore the association of radiotherapy with lung torsion. This may also shed light on the potential mechanism of lung torsion in this case.
